# Prognostic model for lung adenocarcinoma based on experimental drug-resistant cell lines and clinical patients

**DOI:** 10.3389/fmolb.2025.1654426

**Published:** 2025-11-21

**Authors:** Junnan Li, Jiasheng Zhang, Xinyang Zhang, Chengwu Cao, Tianjie Zhou, Fengxian Liu, Liqing Hu, Hang Fai Kwok, Hui Zou

**Affiliations:** 1 Key Laboratory of Study and Discovery of Small Targeted Molecules of Hunan Province, School of Pharmacy, Health Science Center, Hunan Normal University, Changsha, China; 2 Department of Biomedical Sciences, Faculty of Health Sciences, University of Macau, Avenida de Universidade, Taipa, Macao SAR, China; 3 Cancer Centre, Faculty of Health Sciences, University of Macau, Avenida de Universidade, Taipa, Macao SAR, China; 4 MoE Frontiers Science Center for Precision Oncology, University of Macau, Avenida de Universidade, Taipa, Macao SAR, China

**Keywords:** lung cancer, prognosis prediction, EGFR-TKIs resistance, immune microenvironment, single cell RNA-seq (scRNA-seq)

## Abstract

**Objective:**

Despite advances in EGFR-TKIs for lung adenocarcinoma (LUAD), resistance remains a major hurdle. This study aimed to develop a prognostic model integrating immune microenvironment features and *in vitro* resistance mechanisms to predict outcomes and guide therapy.

**Materials and methods:**

erlotinib-, gefitinib-, and osimertinib-resistant HCC827 cell lines were established by exposing them to increasing EGFR-TKIs concentrations. RNA-sequencing was conducted on non-resistant HCC827 and erlotinib/gefitinibresistant cell lines. From the erlotinib-resistant, gefitinib-resistant cell lines and The Cancer Genome Atlas Program-Lung adenocarcinoma (TCGA-LUAD) data, a prognostic risk score model was constructed via Least Absolute Shrinkage and Selection Operator-Cox Proportional Hazards Model (LASSO-COX). Furthermore, immune infiltration was assessed using Gene Set Variation Analysis (GSVA), and single-cell RNA-seq (GSE241934) resolved expression patterns in EGFR-mutant vs. wild-type tumors. *In vitro* validation included RT-PCR in Osimertinib resistant (OR)-HCC827 cells.

**Results:**

A 3-gene (*PPP1R3G, CREG2, LYPD3*) RiskScore were developed. The RiskScore predicted poor survival and resistance across all EGFR-TKI generations, with osimertinib-resistant HCC827 cells showing significant upregulation of signature genes. High-risk patients exhibited immune-suppressive microenvironments (enriched regulatory T cells, depleted mast cells) and distinct scRNA-seq profiles. A nomogram (C-index = 0.7) integrated RiskScore with clinical factors for personalized prognosis.

**Conclusion:**

This model bridges *in vitro* resistance mechanisms with clinical immune landscapes, offering a tool to stratify patients for EGFR-TKIs, immunotherapies, or combinatorial strategies.

## Highlights

- Novel prognostic model: A robust RiskScore model for lung adenocarcinoma (LUAD) was developed using three resistance-associated genes (*PPP1R3G*, *CREG2,* and *LYPD3)*, validated across erlotinib-, gefitinib-, and osimertinib-resistant cell lines and clinical cohorts.
- Immune microenvironment insights: The RiskScore stratifies LUAD patients into distinct immune profiles, with high-risk groups showing elevated regulatory T cells and activated CD4^+^ T cells, suggesting potential resistance to immunotherapies. Single-cell RNA-seq (scRNA-seq) revealed differential gene expression in EGFR-mutant tumors, linking immune evasion to resistance mechanisms.
- Clinical implications: The model stratifies patients into high-risk groups who may be more prone to developing EGFR-TKI resistance, supported by *in vitro* data showing overexpression of the signature genes in osimertinib-resistant cells, which lends preliminary support to its potential clinical relevance.
- Integrated tool: A nomogram combining RiskScore and clinical factors predicts survival and resistance risk, offering a actionable framework for personalized therapy selection.

## 1 Introduction

Lung cancer is still a significant contributor to cancer-related mortality over the world (Siegel et al., 2021). Lung adenocarcinoma, a subtype of non-small cell lung cancer (NSCLC), represents the predominant histological type of lung cancer in humans (Tan et al., 2015). According to World Health Organization (WHO) statistics, adenocarcinoma constitutes roughly 40% of all lung cancer diagnoses. (Yang et al., 2019). Notably, it is the most prevalent lung cancer subtype among non-smokers and is disproportionately common in women as well as in younger individuals (Li et al., 2022). Although the identification of EGFR mutations and the advancement of EGFR tyrosine kinase inhibitors (EGFR-TKIs) have enhanced patient outcomes, the emergence of drug resistance impedes the effectiveness and long-term success of such treatments, presenting a significant challenge in the management of lung adenocarcinoma (Du et al., 2021; Hrustanovic et al., 2013). In light of this, researchers continue to conduct innovative studies aimed at improving the therapeutic efficacy, survival rates, and prognostic outcomes for individuals with lung adenocarcinoma.

Recent research has progressively elucidated the mechanisms underlying resistance to EGFR tyrosine kinase inhibitors (EGFR-TKIs) and established a strong correlation between this resistance and the clinical outcomes of cancer patients following EGFR-TKI therapy (Hayakawa et al., 2013; Kobayashi et al., 2005).After conducting the RNA-sequencing and detecting the differentially expressed genes among erlotinib-resistant cell line, gefitinib-resistant cell line and TCGA-LUAD patients, a prognostic prediction model based on three differentially expressed genes (*PPP1R3G, CREG2, LYPD3*) was established. Besides, the immune landscape of these three genes in LUAD were explored by immune cell infiltration analysis. This prognostic prediction model has been validated in the Gene Expression Omnibus (GEO) database and has the potential to accurately forecast the outcomes for patients with lung adenocarcinoma (LUAD).

Considering all the above facts, this work aims at developing a prognostic prediction model, based on drug resistant genes, so as to provide prognosis information, stratify patients into different risk group, and guide personalized treatment.

## 2 Materials and methods

### 2.1 Establishment of erlotinib-resistant cells and gefitinib-resistant cells

The HCC827 cell line was obtained from the Shanghai Institute for Biological Sciences, which is affiliated with the Chinese Academy of Sciences. These cells were maintained in RPMI-1640 medium, supplemented with 10% fetal bovine serum provided by Gibco™ and sourced from Grand Island, New York. To establish erlotinib-resistant and gefitinib-resistant derivatives of the HCC827 cell line, the parental cells were incrementally exposed to increasing concentrations of either erlotinib or gefitinib. The dosing regimen commenced at 100 nM and culminated at 10 μM (Ikeda et al., 2011; Yamamoto et al., 2010). During the development of the corresponding EGFR-TKI resistance, the medium and drug were replaced twice per week. Subsequent experiments were conducted on these adapted cell lines. HCC827 Osimertinib resistant cell line was kindly given by Prof. Kim Tam from University of Macao.

### 2.2 The 3-(4,5-dimethylthiazol-2-yl)-2,5-diphenyltetrazolium bromide (MTT) assay

MTT reagent (Sigma, Catalog Number: M2128) was used to conducted and evaluate the effectiveness of various treatments. Initially, the EGFR-TKIs resistant cells were plated into a 96-well plate at a density of 5,000 cells per well and allowed to incubate for 24 h. After exposure to PBS or drug treatments for an additional 24 h period, 10 μL of the MTT reagent were introduced into each well, and the cells were incubated for a further 2–4 h. The media were subsequently removed, and 100 μL of dimethyl sulfoxide (DMSO) were added to each well to dissolve the resultant formazan crystals. The optical density (OD) of the wells was then measured at a wavelength of 570 nm using a Thermo Scientific Microplate Reader (Multiskan Spectrum) to quantify the response to treatment. For each treatment condition, we included triplicates (n = 3) in the 96-well plate format, and the entire experiment was repeated three times. IC_50_, or half maximal inhibitory concentration, is a key measure in pharmacology and drug development. IC_50_ is the concentration of a substance (usually a drug or inhibitor) required to inhibit a specific biological or biochemical function by 50%. It’s commonly used to assess the potency of a compound—the lower the IC_50_ value, the more potent the inhibitor.

### 2.3 mRNA extraction and RNA-sequencing

To conduct mRNA extraction and subsequent RNA sequencing, total RNA was isolated utilizing a RNeasy Kit (catalog number 74136, Qiagen, Germany). Complementary DNA (cDNA) libraries were then created using the NEBNext® Ultra™ Directional RNA Library Prep Kit for Illumina® (catalog number E7760, New England Biolabs, Ipswich, MA, USA). These cDNA libraries underwent sequencing on an Illumina Hi-Seq platform (Illumina, San Diego, CA). The initial RNA-sequencing data were assessed with FastQC for quality control. The RNA-sequencing (including data QC, mapping, quantification and differential analysis) for erlotinib drug resistant (EDR) cell line, gefitinib drug resistant (GDR) cell line and HCC827 was conducted by the leading provider of genomic services and solutions company Novogene Co., Ltd. (https://www.novogene.com/amea-en/).

High-quality RNA-sequencing reads from each library were aligned and mapped to the reference genome using STAR v2.6.1day software (developed by the Cold Spring Harbor Laboratory). Total mapping rates of all samples are all larger than 95%.

Genes with expression levels that exhibited a change of more than 2-fold (|log2FoldChange| > 1) and an adjusted p-value (FDR) < 0.05 in the paired samples were considered as upregulated or downregulated. For functional annotation and interpretation of the transcriptome profiles, differentially expressed genes were analyzed through Gene Ontology (GO) analysis. This was performed using the web-based tool DAVID v6.8 (The Database for Annotation, Visualization, and Integrated Discovery, supported by the National Institute of Allergy and Infectious Diseases, part of the NIH). R version 4.3.1 was used for the comparative analysis of RNA-sequencing. Differential expression genes (DEGs) analysis of LUAD in TCGA was performed by R package Deseq2 and raw count was used as input. DEGs analysis between two risk group in GEO data was conducted by R package “Limma”. Fragments per kilobase of transcript per million mapped reads (FPKM) was used for survival anlysis and gene expression in cross-sample comparison.

### 2.4 Data acquisition

The transcriptome profiles along with the corresponding clinical data for 50 normal and 504 lung adenocarcinoma (LUAD) samples were downloaded from The Cancer Genome Atlas (TCGA) database (https://portal.gdc.cancer.gov/). The RNA expression data and clinical information were download and accessed by the project name TCGA-LUAD and Experimental Strategy RNA-Seq. In addition, the microarray data and related clinical details for 11 normal and 57 LUAD samples were obtained from the Gene Expression Omnibus (GEO) under accession number GSE116959, using platform GPL17077 (https://www.ncbi.nlm.nih.gov/geo/query/acc.cgi?acc=GSE116959). Also, we retrieved microarray data for 442 LUAD samples from the GEO dataset with accession number GSE72094, which is based on platform GPL15048, accessed on the 27 May 2024 through the GEO website (https://www.ncbi.nlm.nih.gov/geo/query/acc.cgi?acc=GSE72094). The single-cell RNA sequencing (scRNA-seq) data GSE241934 (https://www.ncbi.nlm.nih.gov/geo/query/acc.cgi?acc=GSE241934, download in 27 March 2025) comprises of 11 resected tumors from earlystage EGFR-mutant patients as well as 34 tumors which were all confirmed wildtype lung adenocarcinoma (LUAD) or adenosquamous carcinoma (AdSqC). Candidate genes were analyzed in LUAD patients harboring EGFR mutation in (L858R, exon 19 deletions [Exon19del], and exon 20 insertions [Exon20ins]), compared with LUAD patients with wild type EGFR. R package “Seurat” V5 was used to filter and process scRNA-seq data. R package ‘singleR’ was conducted for cell cluster annotation, which is a commonly used computational framework for the annotation of scRNA-seq by reference to bulk transcriptomes (Xin et al., 2024; Aran et al., 2019). TCGA-LUAD cohort was used as training dataset and the other three independent dataset GSE 116959, GSE72094 and GSE241934 were used as validation datasets, to delineate mutation-specific transcriptional signatures.

### 2.5 GO and KEGG functional enrichment analyses

Functional enrichment analyses using Gene Ontology (GO) and Kyoto Encyclopedia of Genes and Genomes (KEGG) were performed to investigate the biological processes associated with differentially expressed genes. This analysis was conducted utilizing R statistical software packages, including “clusterProfiler,” “org.Hs.e.g.,.db,” “enrichplot,” “ggplot2,” and “GOplot.” The GO analysis provided insights into three main categories: cellular component (CC), biological process (BP), and molecular function (MF).

### 2.6 LASSO-COX dimension reduction analysis

The LASSO-COX dimension reduction method was utilized to analyze data, employing the “glmnet” (Lasso and Elastic-Net Regularized Generalized Linear Models) and “survival” packages within the R programming environment. Lambda is regularization parameter, controlling the amount of shrinkage. We used Cox regression model with the LASSO to achieve shrinkage and variable selection simultaneously. Ten-fold cross validation was used to determine the optimal value of λ. This method divided the TCGA-LUAD RNA-sequencing data into 10 subsets (folds). For each fold, the model was trained on k-1 folds and validate it on the remaining fold and the performance metric like partial likelihood deviation was recorded in a range of λ value. Then the partial likelihood deviation was averaged across all folds for each λ. In the training process, a subset of variables was identified by shrinking the coefficients of less important variables into zero. We selected λ.min for our final model. We note that the more parsimonious λ.1se criterion resulted in a null model (zero genes). Given that the 3-gene signature at λ.min was highly predictive and successfully validated externally, we proceeded with this model to identify a biologically and clinically relevant signature.The optimal λ value lambda.min (λ.min) for our research was identified as the one that corresponded to the lowest partial likelihood deviance and minimum mean cross-validated error. Ultimately, based on the optical optimalλ (λ.min = 0.09785), we identified three genes of interest along with their respective coefficient: *PPP1R3G* (0.07797704), *CREG2* (0.04387373) and *LYPD3* (0.02302433). The risk score for each patient was derived using the following formula:

$$\begin{array}{ll} \text{risk}\;\text{score} & =\left(\text{expression}\;\text{of}\;PPP1R3G\times \text{coefficient}\;\text{for}\;PPP1R3G\right)\\ & \;+\left(\text{expression}\;\text{of}\;CREG2\times \text{coefficient}\;\text{for}\;CREG2\right)\\ & \;+\left(\text{expression}\;\text{of}\;LYPD3\times \text{coefficient}\;\text{for}\;LYPD3\right) \end{array}$$

In this formula, “expression of gene” refers to the gene’s expression level, and “coefficient for gene” is its coefficient corresponding λ.min.

### 2.7 Nomogram construction and time-dependent AUC

A nomogram analysis was developed within the training cohort using the Regression Modeling Strategies (rms) package in R. This nomogram is bifurcated, with the upper section serving as a scoring guide and the lower section as a predictive tool. The nomogram enables the precise prediction of the 1-, 2-, 3-, 5-, and 10-year survival for patients with LUAD, based on the cumulative points of each contributing factor. The accuracy of the nomogram in predicting overall survival (OS) was validated in the validation group. Calibration curves and C-Index values were employed to assess and quantify the precision of these survival predictions. Time-dependent AUC was calculated using the “timeROC” R package at 1, 2, and 3-year time points. Bootstrap C-index with 1000 resamples was performed using the “boot” package.

### 2.8 Immune cell infiltration analysis

We utilized Gene Set Variation Analysis (GSVA) to analyze the immune microenvironment within LUAD tumors (Li, 2017). This technique enables the identification of 28 distinct immune cell populations, such as seven subtypes of T cells, plasma cells, naive and memory B cells (Charoentong et al., 2017). We depicted the variations in immune cell composition between high-RiskScore and low-RiskScore groups through bar plots. The GSVA generates normalized scores ranging from 0–1, representing the abundance of the immune cell population. In subsequent analyses aimed at identifying differences in immune cell infiltration levels between these two groups, only samples with a p-value of less than 0.05 were taken into account (Systematic RNA, 2009).

### 2.9 Statistical analysis

Statistical analyses were conducted utilizing R (https://www.r-project.org/, v3.5.0), available at R Project, SPSS software version 25.0 from IBM, headquartered in Chicago, IL, and GraphPad Prism version 8.0, which is a product of La Jolla, CA. The prognostic significance was assessed through Kaplan-Meier survival analysis and COX proportional hazards modeling. Gene Set Enrichment Analysis (GSEA) was conducted using the GSEA package accessible through the Broad Institute’s website at GSEA (http://software.broadinstitute.org/gsea/index.jsp), while Gene Ontology (GO) and KEGG (Kyoto Encyclopedia of Genes and Genomes) analysis was carried out using the clusterProfiler package. A p-value of less than 0.05 was set as the threshold for statistical significance across all methods.

## 3 Results

### 3.1 Differential expressed genes (DEGs) among EGFR-TKIs resistant cells and LUAD patients

**FIGURE 1:**
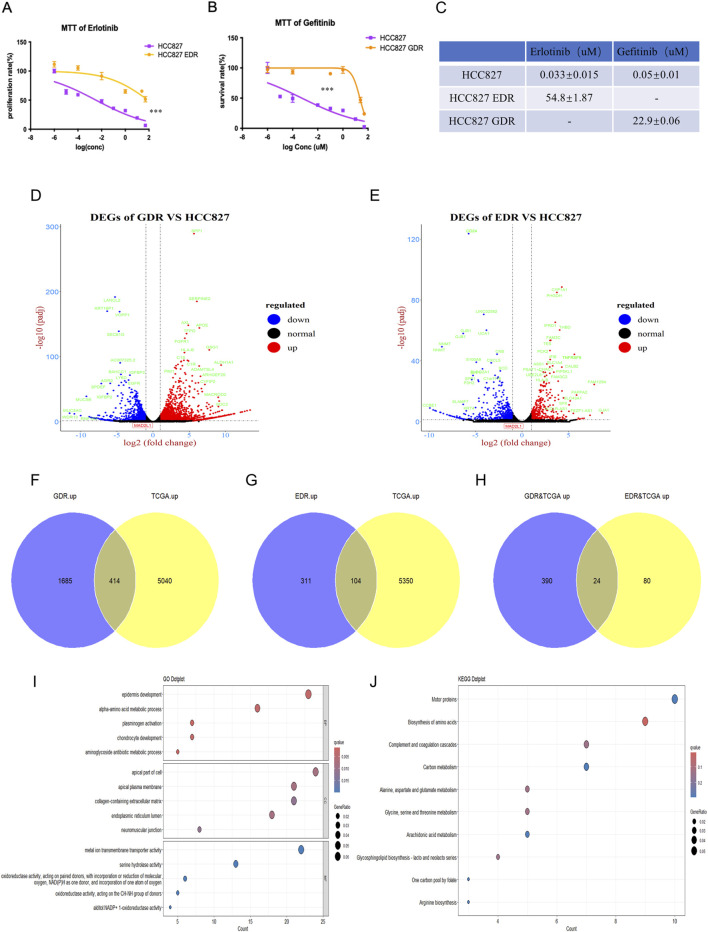
Establishment of erlotinib and gefitinib resistance cell line. **(A–C)** The IC50 of erlotinib and gefitinib were detected in erlotinib-resistant cell line, gefitinib-resistant cell line and paternal HCC827 cell line via MTT, respectively. ***P < 0.001, by Student’s t-test. **(D,E)** Differential expression genes were detected via RNA-sequencing in gefitinib-resistant cell line and erlotinib-resistant cell line. **(F–H)** Venn plot showed the overlap of upregulated genes between gefitinib/erlotinib resistant cell line and LUAD patients’ tissues in TCGA. **(I,J)** GO and KEGG analyses revealed the enriched pathways among the upregulated differentially expressed genes in GDR&TCGA and EDR&TCGA.

To simulate the clinical development of drug resistance and enhance the potency of EGFR-TKIs therapies, we developed EGFR-TKIs-resistant HCC827 cell lines. This was achieved by culturing the cells in increasing doses of erlotinib and gefitinib, resulting in the HCC827 EDR (erlotinib-resistant cell line) and HCC827 GDR (gefitinib-resistant cell line), respectively. As shown in Figure 1A, HCC827 EDR cells were less sensitive to erlotinib treatment alone than parental HCC827 cells. Additionally, the IC50 value for erlotinib was significantly higher in the HCC827 EDR cells (54.8 ± 1.87 uM) than parental HCC827 cells (0.033 ± 0.015 uM) (Figure 1C). The phenomenon also be observed with the HCC827 GDR cells (Figures 1B,C). These results collectively indicate that we have successfully established EGFR-TKI-resistant cell lines, which will serve as valuable models for further investigation.

Following the development of drug-resistant cell lines, RNA-sequencing was per-formed for the parental HCC827 cells, HCC827 EDR cells and HCC827 GDR cells. We then conducted a comparative analysis of the RNA sequencing data between the parental HCC827 cells and each of the resistant cell lines—HCC827 EDR and HCC827 GDR—to discern the genes that were differentially expressed. Using a threshold of FDR<0.05 and |log2FC| > 1 (2 fold change),the volcano plot analysis of HCC827 GDR cells uncovered 3264 differentially expressed genes (DEGs), with 2099 genes upregulated and 1165 genes downregulated (Figure 1D). Meanwhile, the HCC827 EDR cells exhibited a different pattern, with 415 genes upregulated and 514 genes downregulated (Figure 1E). To dissect and pinpoint the central genes associated with resistance to EGFR-TKIs and the prognostic indicators of lung cancer, a Venn diagram analysis was employed. This method was utilized to identify genes that were co-regulated across both the experimentally derived drug-resistant lung cancer cells and samples from clinical patients. The Venn diagram analysis demonstrated a shared upregulation of 414 genes in both the HCC827 GDR cells and tumor tissues from lung adenocarcinoma (LUAD) patients (Figure 1F). Additionally, it revealed that 104 genes were commonly upregulated in the HCC827 EDR cells and LUAD tumor tissues (Figure 1G). Only 24 genes were found to be concurrently upregulated in both drug-resistant cells and LUAD tumor tissues, as depicted in Figure 1H. Additionally, Gene Ontology (GO) and Kyoto Encyclopedia of Genes and Genomes (KEGG) analyses were performed on the total 494 upregulated genes in drug-resistant cells and LUAD tumor tissues. These analyses indicated an enrichment of genes associated with amino acid biosynthesis and metabolism, which may play a role in tumor progression and the development of acquired drug resistance, as illustrated in Figures 1I,J.

### 3.2 Model construction based on the (DEGs) among EGFR-TKIs resistant cells and LUAD patients

**FIGURE 2:**
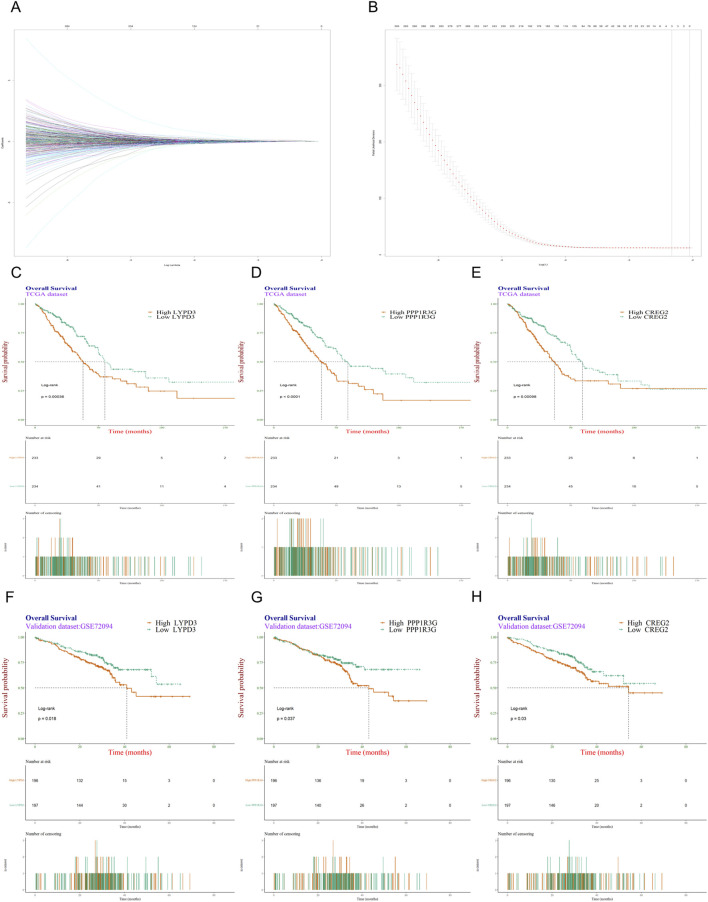
Screening and validation of genes most associated with resistance to EGFR-TKIs. **(A)** Trace plot of coeffecient fit by LASSO-Cox: each curve corresponding to a variable; **(B)** Cross-validation curve for the LASSO-Cox regression model. The left dashed line indicates λ.min, and right dashed line indicates λ.1se; **(C–E)** Kaplan-Meier survival analysis was conducted on the TCGA dataset to evaluate the influence of three individual genes, LYPD3, PPP1R3G, and CREG2, on patients’ overall survival. **(F–H)** The KM survival analysis was validated in another independent dataset GSE72094.

To establish a prognostic model for LUAD patients, LASSO-COX dimension reduction analysis was conducted based on those 494 DEGs. The cross-validation plot shows the deviance across log(λ) values. The left dashed line indicates λ.min, which selected a 3-gene signature. The right dashed line indicates λ.1se, which resulted in a null model (zero genes) (Figures 2A,B). Finally, three candidate genes (*PPP1R3G*, *CREG2* and *LYPD3*) and their corresponding lambda values were used to calculate the RiskScore for each patient. All three of these were newly identified biomarkers for lung cancer and EGFR-TKIs resistance. The median RiskScore (0.19182) of the training database was set as the cutoff value.

**FIGURE 3:**
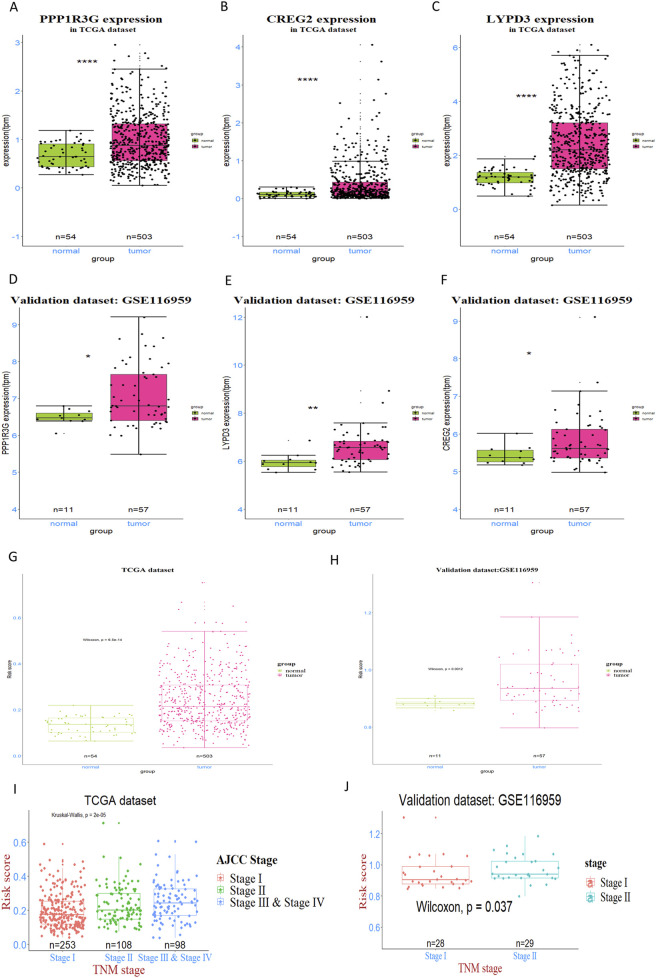
Expression analysis and validation of three prognostic genes in LUAD patients. **(A–C)** The expression levels of three genes—PPP1R3G, CREG2, and LYPD3—were detected and compared between tumor tissues and normal tissues in TCGA dataset. **(D–F)** This pattern was further validated in GSE116959 dataset. **(G, H)** the RiskScore, derived from the expression of the genes PPP1R3G, CREG2, and LYPD3, was assessed and compared between tumor and normal tissues in both the training and validation datasets **(I, J)** The Kruskal–Wallis test was applied to identify overall differences in risk scores across various tumor stages, while the Wilcoxon test was used for pairwise comparisons to pinpoint specific stage-related disparities.

To explore the prognostic prediction value of *PPP1R3G*, *CREG2* and *LYPD3* in LUAD patients, we conducted Kaplan-Meier analyses based on the TCGA and GEO databases respectively. Patients with higher expression of *PPP1R3G*, *CREG2* and *LYPD3* all had significantly shorter overall survival compared with those with lower expression in the TCGA database (Figures 2C–E). In addition, the survival rate of *PPP1R3G*, *CREG2* and *LYPD3* was verified in the GEO database (Figures 2F–H). Besides, the individual gene expression analyses of *PPP1R3G*, *CREG2*, and *LYPD3* indicated that the expression levels of all three genes were significantly elevated in lung tumor tissues as compared to normal tissues. This overexpression was consistently observed in both the training and validation datasets (Figures 3A–F). To validate the translational relevance of our signature, we investigated the protein expression of our three genes using the CPTAC-LUAD proteomic dataset. This analysis revealed that the protein levels of both LYPD3 and PPP1R3G were significantly elevated in lung adenocarcinoma tissues compared to matched normal adjacent tissues (p < 0.05 for both; Reference to the supplementary S1A,B). The protein for CREG2 was not detected in this cohort. These results demonstrate that the prognostic signal from our transcriptomic signature is reflected in actual protein abundance changes for the majority of its components in patient tumors, strengthening its biological and clinical relevance.

These findings collectively indicate that the expression levels of *PPP1R3G*, *CREG2*, and *LYPD3* are strong prognostic indicators for LUAD patients, suggesting their potential utility in predicting outcomes of EGFR-TKI treated patients and guiding personalized treatment decisions.

### 3.3 Relationship between RiskScores of the prognostic signature and clinical-pathologic characteristics

In order to figure out the relationship between RiskScores and clinical-pathologic characteristics, the correlation between the RiskScore and various clinical and pathological factors was further examined. It was found that the RiskScore was significantly higher in tumor tissues compared to normal tissues in both TCGA and GEO patient cohorts (Figures 3G,H). In addition, the RiskScore exhibited a moderate, albeit slight, increase in patients with more advanced tumor stages, as observed in both the training and validation databases (Figures 3I,J). Nonetheless, in both the training and validation datasets, no association was observed between the RiskScore and factors such as gender, age and race. These findings suggest that the RiskScore is primarily associated with tumor biology rather than demographic factors, highlighting its potential as a valuable biomarker for predicting LUAD prognosis and guiding personalized treatment strategies.

### 3.4 Relationship between RiskScore and patients’ survival

**FIGURE 4:**
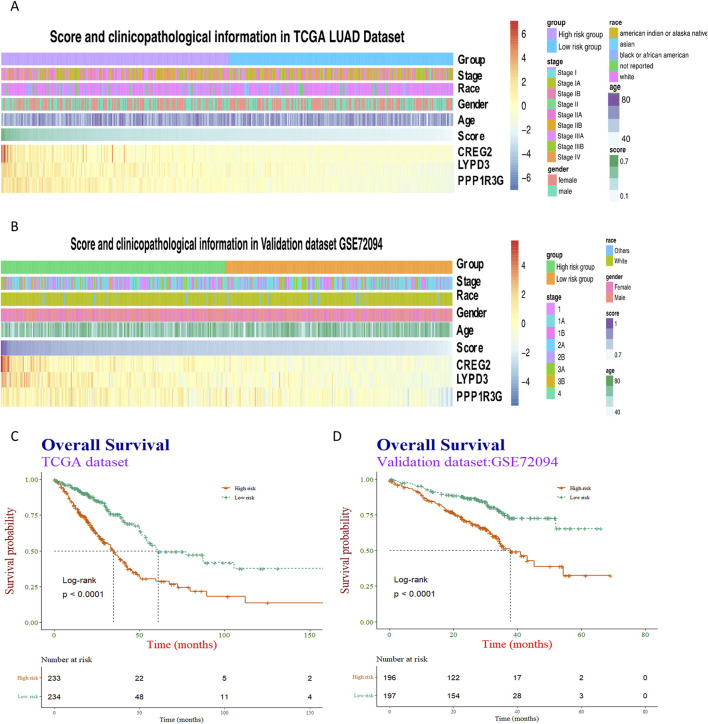
Heatmap visualization and survival analysis of clinical-pathological factors and gene expression in cancer cohorts. **(A,B)** Clinical-pathological factors and the expression of three representative genes—PPP1R3G, CREG2, and LYPD3—were visualized in heatmaps arranged by descending risk score, both in the training dataset and the validation dataset. **(C,D)** The association between the risk score and patients’ overall survival was then examined using Kaplan-Meier survival curves, with separate analyses conducted for the training and validation cohorts.

Then, patients were further categorized into distinct RiskScore groups and demon-strated comparable profiles in terms of clinical and pathological traits, mirroring the patterns observed in the training dataset (Figures 4A,B). However, no significant different in terms of clinical and pathological traits between the high-RiskScore group and low-RiskScore group.

Subsequently, Kaplan-Meier analyses were performed taking into account the RiskScore. The prognostic models exhibited enhanced predictive accuracy for overall survival and progression-free survival across both the training and validation datasets (Figures 4C,D). These results indicate that the RiskScore is a powerful predictor of clinical outcomes in LUAD.

### 3.5 The RiskScore is closely related to cell division and DNA metabolism

**FIGURE 5:**
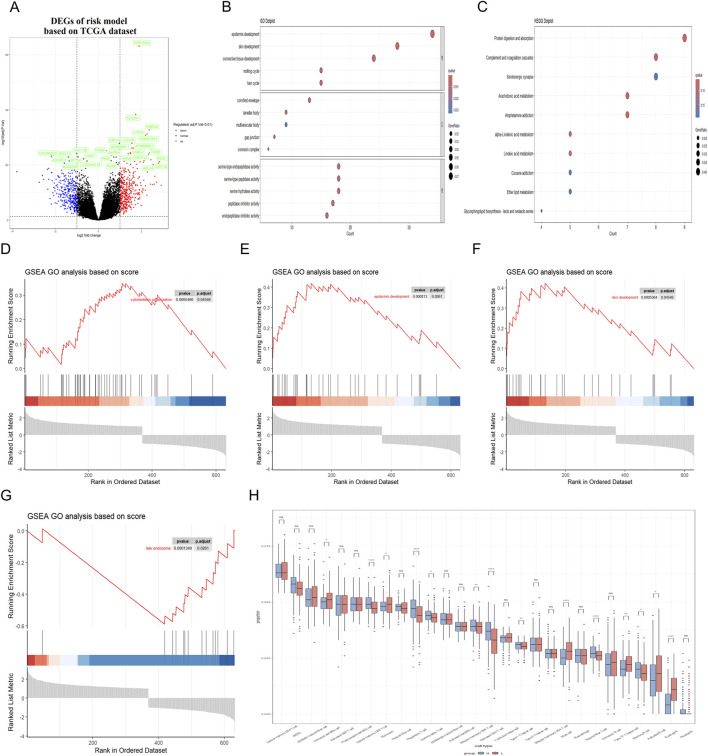
Correlation between RiskScore and biological functions in training dataset. **(A)** Volcano plot was utilized to illustrate the differentially expressed genes (DEGs) between the high-risk and low-risk groups in the TCGA dataset. **(B,C)** GO and KEGG analyses were conducted to identify the enriched signaling pathways associated with these DEGs. **(D–G)** GSEA revealed significant upregulation of pathways related to cytoskeleton organization, cell cycle processes, epidermal and skin development; Besides, the late endosome pathway was found to be significantly downregulated. **(H)** Immune cell infiltration analysis was conducted to demonstrate a close association between DEGs and various immune cells.

In an effort to uncover the biological functions and pathways that correlate with the RiskScore, a series of analytical methods were employed. Initially, genes with the strongest ties to the RiskScore were identified. We conducted a comparative analysis of the differentially expressed genes between the high-RiskScore group and low-RiskScore group, and the volcano plot analysis uncovered 731 differentially expressed genes (DEGs), with 405 genes upregulated and 326 genes downregulated (Figure 5A).

**FIGURE 6:**
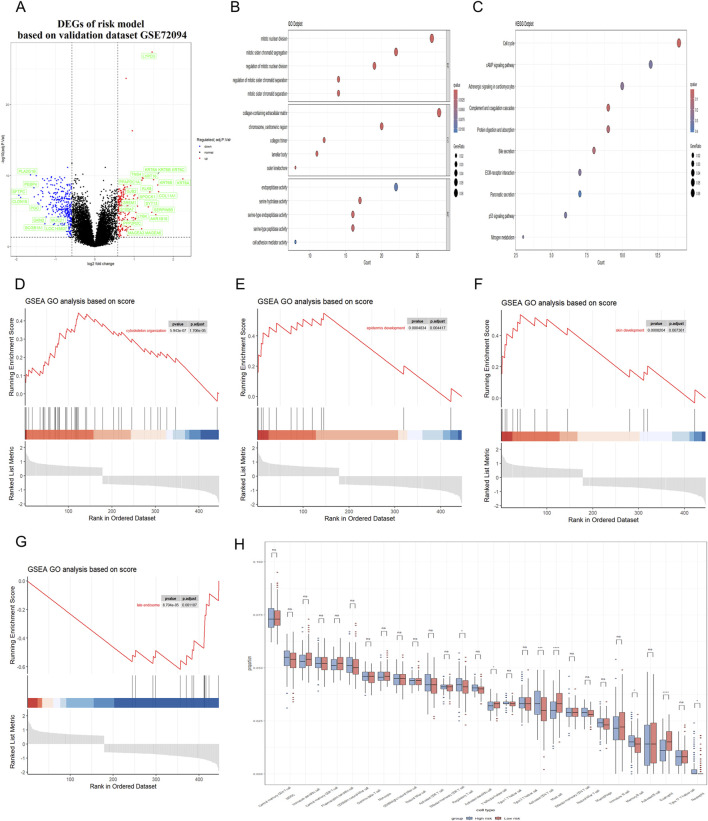
Correlation between RiskScore and biological functions in validation dataset. **(A)** Volcano plot was utilized to illustrate the differentially expressed genes (DEGs) between the high-risk and low-risk groups in the GEO dataset. **(B,C)** GO and KEGG analyses were conducted to identify the enriched signaling pathways associated with these DEGs. **(D–G)** GSEA revealed significant upregulation of pathways related to cytoskeleton organization, cell cycle processes, epidermal and skin development; Besides, the late endosome pathway was found to be significantly downregulated. **(H)** Immune cell infiltration analysis was conducted to demonstrate a close association between DEGs and various immune cells.

Subsequently, Gene Ontology (GO) and Kyoto Encyclopedia of Genes and Genomes (KEGG) were utilized to conduct a functional enrichment analysis on this refined set of genes. Additionally, Gene Set Enrichment Analysis (GSEA) was applied to further elucidate the biological significance behind the RiskScore. The GO analysis showed that the RiskScore was closely related to the connective tissue development and serine-type peptidase activity (Figure 5B). The KEGG analysis showed that the RiskScore was closely related to the protein digestion and absorption signaling pathway (Figure 5C). Furthermore, the close association of the RiskScore with key biofunctions and signaling pathways—such as cytoskeleton organization, late endosome function, skin and epi-dermal development—was corroborated through GSEA analysis of data from the TCGA databases (Figures 5D–G). GSEA revealed significant upregulation of pathways associated with cytoskeleton organization, epidermal development, and skin development. In contrast, the late endosome pathway exhibited significant downregulation. The similar finding could also be observed in the validation datasets (Figures 6A–G).

### 3.6 Association analysis of RiskScore with tumor immune microenvironment characteristics and EGFR mutation status

In addition, the presence of immune cells within tumors significantly influences both neoplastic progression and the effectiveness of therapies designed to combat cancer. Herein, we conducted an analysis to determine the extent of immune cell infiltration in both TCGA database and GEO database. Our findings indicated that in the high RiskScore group, there was an increase in the infiltration of regulatory T cell, Memory B cell and activated CD4 T cell. Conversely, in the low RiskScore group, a higher level of infiltration was observed for mast cell and eosinophil with these differences being statistically significant (p < 0.05) (Figure 5H). The similar finding could also be observed in the validation GEO database, strongly suggesting that high-RiskScore patients may exhibit reduced responsiveness to immunotherapies due to their immunosuppressive microenvironment (Figure 6H). However, the tumor immune microenvironment is an intricate system, and it is premature to categorically determine whether the presence of immune cell infiltration is beneficial or harmful to patients. The multifaceted nature of these cells within the tumor microenvironment necessitates further investigation to elucidate their precise roles. Various studies are essential to clarify the implications of these immune cells on patient outcomes. These results suggest that the high RiskScore group and low RiskScore group possess distinct immune profiles and exhibit varied reactions to immunotherapeutic interventions that target distinct immune checkpoints.

**FIGURE 7:**
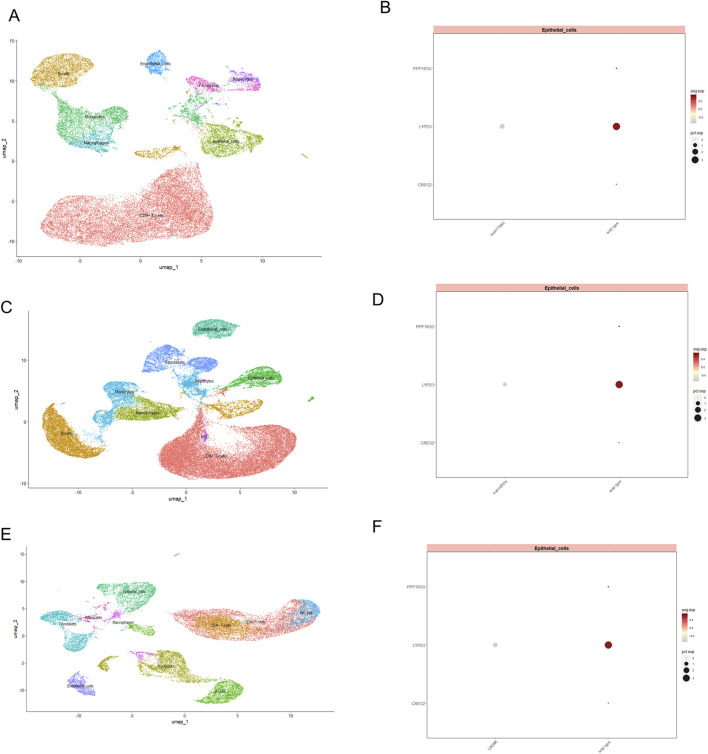
The expression of three model genes in EGFR-TKI resistant LUAD patients revealed by single cell transcriptomics profiling. UMAP visualization of single-cell RNA-seq data, illustrating the distribution of distinct cellular populations in tumor tissue from LUAD patients with exon19del **(A)**, exon20ins **(C)** and L858R **(E)**. Each cluster represents a transcriptionally distinct cell type, with epithelial cells prominently marked. Dot plot displaying the expression levels of PPP1R3G, LYPD3, and CREG2 in epithelial cell subsets between wild type and patients with exon19del **(B)**, exon20ins **(D)** and L858R **(F)** mutation. Dot size indicates the proportion of cells expressing each gene, while color intensity reflects the average expression level.

Moreover, we conducted single-cell RNA sequencing (scRNA-seq) analysis of GSE241934 dataset to reveal distinct expression patterns of *LYPD3*, *PPP1R3G*, and *CREG2* between EGFR-mutant and wild-type LUAD patients. We note that this cohort consists of LUAD patients who received neoadjuvant immunochemotherapy, a potential confounder for gene expression. With this important caveat, we observed that these three genes exhibited significant downregulation in tumor cells from EGFR-mutant patients (L858R/Exon19del/Exon20ins) compared with non-mutant counterparts (Figures 7A–F), though we cannot rule out that this expression pattern was influenced by the prior therapy. This observation appears paradoxical to our cell line model where these genes were upregulated in acquired resistance. However, this discrepancy may reflect fundamental differences in resistance mechanisms and could be explained by several hypothesis: 1. We speculate that the cell line model simulates acquired resistance through progressive drug selection, whereas clinical samples in GSE241934 represent intrinsic resistance or early treatment-naive mutations. 2. Alternatively, the clinical cohort received neoadjuvant immunochemotherapy, which may induce immunomodulatory changes that suppress these genes’ expression. These findings collectively suggest that these genes are closely associated with acquired resistance (as shown *in vitro*). Their expression patterns in clinical settings may be influenced by additional layers of tumor heterogeneity, treatment interventions, and immune system interactions that require further investigation. To address the potential confounding effect of neoadjuvant immunochemotherapy in the single-cell cohort (GSE241934), we investigated the relationship between our 3-gene signature and treatment response. We found no significant difference in the signature risk score between patients who achieved a Major Pathological Response (MPR) and those who did not (p = 0.46; Reference to supplementary S2A). This indicates that the signature is not a direct predictor of response to this regimen. Despite this, the signature retained its power to stratify patients by overall survival, suggesting it captures fundamental aspects of tumor biology and intrinsic aggressiveness that are independent of the response to immunochemotherapy.

### 3.7 The individualized prediction model showed robust predictive accuracy

**FIGURE 8:**
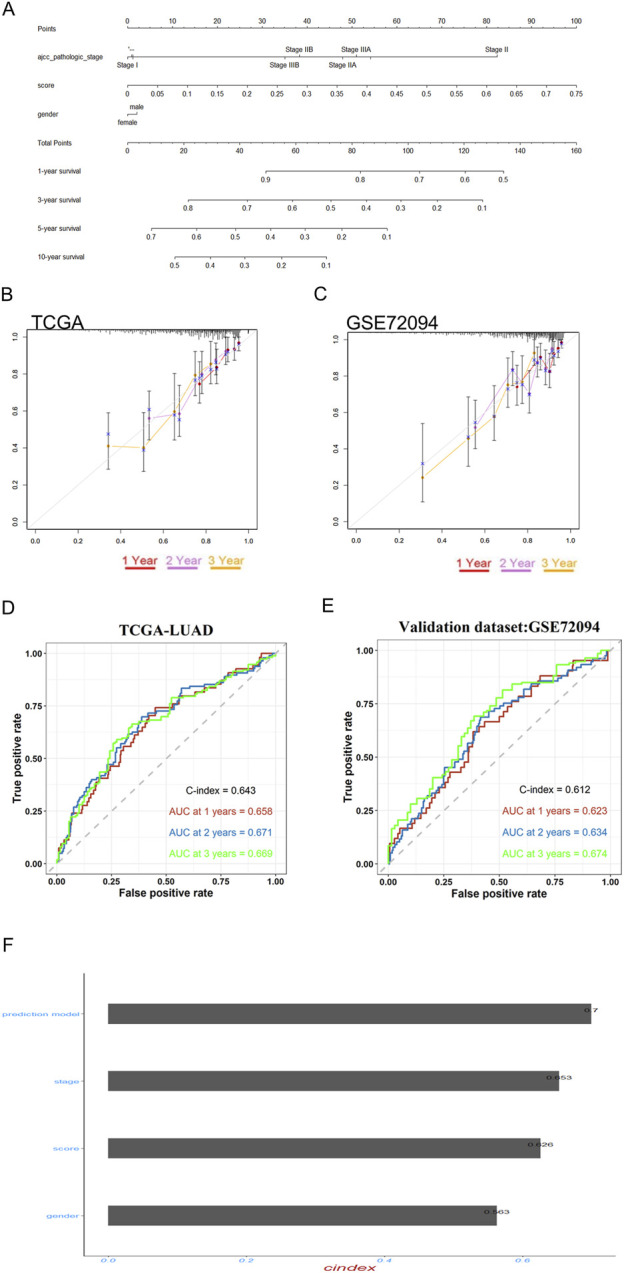
Prognostic model validation and personalized prediction of LUAD patient survival post-EGFR-TKI treatment. **(A)** The nomogram accurately predicted the 1-, 3-, 5-, and 10-year overall survival (OS) times for lung adenocarcinoma (LUAD) patients following treatment with gefitinib and erlotinib. **(B,C)** Calibration plots were employed to assess the concordance between the predicted and actual OS rates at 1-, 2-, and 3-year intervals for both the training and validation datasets, demonstrating the model’s reliability. **(D,E)** time-dependent ROC and bootstrap C-index analyses of the 3-gene signature in TCGA-LUAD and external validation cohort GSE72094 **(F)** The predictive efficacy of the personalized prognostic model, along with the individual risk score and clinical prognostic factors for LUAD patients’ OS, was evaluated using the concordance index (C-Index).

To enhance the practicality of the prognostic prediction model in clinical settings, a personalized prediction model was developed. A personalized model for predicting Progression-Free Survival (PFS) was developed, incorporating a set of independent predictive factors. These included the RiskScore, gender and pathologic stage. Figure 8A illustrates that the individualized prediction model can be used to estimate the tumor recurrence probability for Lung Adenocarcinoma (LUAD) patients at various time points, including 1-, 3-, 5-, and 10-year post-treatment. The calibration curve, which compares the nomogram predictions with actual outcomes, demonstrates a good match in both the training and validation datasets, as depicted in Figures 8B,C, suggesting high predictive accuracy. Furthermore, to quantify the precision of the model’s predictive accuracy, we computed the C-index with a 95% confidence interval via 1000 bootstrap resamples. The prognostic performance of the 3-gene signature was consistent across both the training and external validation cohorts. In the training set, the model achieved a C-index of 0.643 (95% CI: 0.594–0.687), which was closely replicated in the independent validation set with a C-index of 0.612 (95% CI: 0.556–0.671). Similarly, time-dependent AUC values ranged between 0.62 and 0.67 in both cohorts at key time points (Figures 8D,E). These results indicate that the signature provides a modest but stable and generalizable level of discriminative ability.The C-index of this nomogram model was 0.7, which is higher than any other prediction model (Figure 8F). These findings collectively underscore the potent predictive capability of our developed model. To evaluate the potential of the RiskScore as a predictive biomarker, we assessed its association with clinical response to therapy. In the GSE241934 cohort of patients treated with neoadjuvant immunochemotherapy, we found no significant difference in the RiskScore between patients who achieved a Major Pathological Response (MPR) and those who did not (p = 0.46; supplementary S2A). This indicates that the RiskScore is not predictive of response to this treatment regimen. Its validated utility remains its significant association with overall survival, establishing it as a prognostic biomarker.

### 3.8 Validation of the prognostic model in osimertinib-resistant cell lines

**FIGURE 9:**
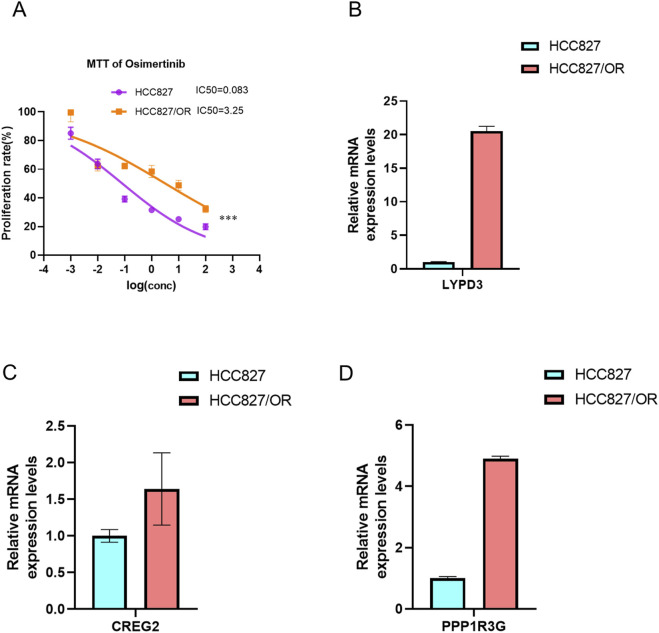
Establishment of Osimertinib resistance cell line. The IC50 of Osimertinib were detected in osimertinib-resistant cell line and paternal HCC827 cell line via MTT,***P < 0.001, by Student’s t-test. **(A)** The bar graph showed the expression of three drug resistant associated genes LYPD3 **(B)**, CREG2 **(C)** and PPP1R3G **(D)** between osimertinib-resistant cell line and paternal HCC827 cell line, detected by RT-PCR.

To further validate the reliability of our prognostic model, we extended our analysis to third-generation EGFR-TKI (osimertinib)-resistant cell lines. Specifically, we established an osimertinib-resistant HCC827 cell line (OR-HCC827) by progressively exposing parental HCC827 cells to increasing concentrations of osimertinib. Consistent with our observations in erlotinib- and gefitinib-resistant models, the OR-HCC827 cells exhibited significantly higher IC50 values compared to the parental HCC827 cells (Figure 9A), confirming the successful development of osimertinib resistance.

Subsequently, RT-PCR analysis revealed that the expression levels of *LYPD3*, *PPP1R3G*, and *CREG2* were markedly upregulated in OR-HCC827 cells relative to the parental line (Figures 9B–D). These findings corroborate the robustness of our prognostic signature across multiple generations of EGFR-TKIs. The consistent overexpression of these biomarkers in osimertinib-resistant cells further supports their potential utility in stratifying high-risk LUAD patients.

## 4 Discussion

The discovery of EGFR-activating mutations in a subset of lung cancer patients led to the use of EGFR tyrosine kinase inhibitors (EGFR-TKIs) to treat non-small cell lung cancer (NSCLC) with these specific mutations (Kobayashi et al., 2005; Bean et al., 2007; Ninomiya et al., 2018). While EGFR-TKIs have significantly improved outcomes for these patients, resistance to these drugs is a common issue. Furthermore, lung adenocarcinoma (LUAD) is characterized by a poor prognosis and a lack of effective screening techniques, contributing to a low rate of successful clinical treatment (Lee et al., 2020; Altintas and Tothill, 2013). Consequently, there is a pressing need to develop innovative biomarkers that can accurately predict the prognosis of LUAD after EGFR-TKIs treatments.

In the current study, we embarked on a multifaceted approach to tackle the challenge of acquired resistance to EGFR-TKIs in LUAD. Initially, we developed EGFR-TKIs resistant cell lines and this *in vitro* model provided a valuable platform for identifying genes that are crucial in the resistance mechanism. The discovery of 494 co-upregulated genes in resistant cells and LUAD patient samples represents a significant step forward in understanding the molecular underpinnings of resistance. Through bioinformatics analysis, we identified pathways related to amino acid biosynthesis and metabolism as being central to the resistant phenotype. Amino acid metabolism is intricately linked to drug resistance in cancer cells, where certain amino acids, such as glutamine and serine, can fuel metabolic pathways that neutralize drug effects or promote repair mechanisms, leading to decreased treatment efficacy. These results have facilitated the identification of potential resistance-related markers and have led to the development of a prognostic model that shows promise in guiding clinical treatment strategies. Then, the LASSO-COX method for dimensionality reduction was employed to pinpoint an optimal set of prognostic indicators. This analysis led to the discovery of a 3-gene signature—comprising *PPP1R3G*, *CREG2*, and *LYPD3*—that is characteristic of the resistant lung cancer cells. We acknowledge that the LASSO λ.1se criterion, which favors maximum parsimony, did not select any genes, indicating the challenge of deriving a highly stable signature from this gene set. However, the external validation of our λ.min-derived 3-gene signature confirms its robust prognostic value, suggesting these genes capture a meaningful biological signal related to prognosis in lung adenocarcinoma.

Researchers have identified several genes associated with cancer progression and treatment resistance. *PPP1R3G*, encoding a regulatory subunit of protein phosphatase 1 (PP1), plays a role in cellular processes like cell division and signal transduction (Shi et al., 2023; Zhuo et al., 2021). Its dysregulation may contribute to cancer therapy resistance by helping cancer cells evade drug effects. *CREG2*, involved in cellular respiration and energy metabolism, has potential indirect effects on cancer development. The CREG2 gene is implicated in Lung Adenocarcinoma (LUAD), where its high expression is linked to poor prognosis and advanced tumor stages. CREG2’s role in cancer is complex, with some studies suggesting it may have a pro-cancer effect by influencing stromal cells and proliferation, while other research has identified it as a potential prognostic biomarker that could help in predicting patient outcomes and guiding personalized treatment strategies. (Kunita et al., 2002; Min et al., 2025; Hao et al., 2025). *LYPD3*, encoding a lysozyme-like protein, is implicated in immune response and cell differentiation (Gruet et al., 2020; Wang et al., 2017). It may modulate immune cell activity against tumors, with its expression linked to distinct immune profiles and responses to immunotherapy in different risk groups (Xin et al., 2024; Hu et al., 2020). While *LYPD3’s* role in cancer is becoming more defined, additional studies are needed to confirm its involvement in cancer development or progression.

Then, a novel RiskScore-based nomogram was constructed to predict the prognosis and resistance of LUAD following curative resection. The nomogram, incorporating the RiskScore, patient age, gender and pathologic stage successfully identify patients at high risk of acquired resistance. The nomogram serves as a visual aid for clinicians to estimate the risk of acquired resistance and tailor treatment strategies accordingly, thereby enhancing the precision of patient care. Additionally, the successful validation in OR-HCC827 cells (Figures 9B–D) confirms these genes' potential role in resistance mechanisms spanning all generations of EGFR-TKIs. Our study successfully derives and validates a 3-gene prognostic signature for lung adenocarcinoma, rooted in the biology of experimentally-defined drug resistance. The model demonstrated consistent and statistically significant performance in both the discovery and external validation cohorts, with C-indices consistently around 0.65. While the discriminative accuracy is modest, this level of consistency strongly argues against overfitting and confirms that the signature captures a real and reproducible biological signal relevant to patient outcomes. It is important to note that such compact transcriptomic signatures often explain a portion of the prognostic variance; their clinical utility may ultimately lie in being integrated with established clinical variables like stage or performance status to build more powerful composite models. The independent validation of LYPD3 and PPP1R3G protein overexpression in LUAD tumors via the CPTAC database provides crucial support for our findings. It moves our signature beyond a transcriptomic correlation to a finding with direct implications for the tumor’s functional proteomic state. The absence of CREG2 data in this resource highlights a limitation but does not diminish the collective evidence supporting the signature’s biological plausibility.

Additionally, we also examined the relationship between RiskScore and both Tumor Immune Microenvironment (TME) characteristics and EGFR mutation status. Our immune infiltration analyses revealed that high-RiskScore tumors exhibit an immunosuppressive profile characterized by elevated regulatory T cells (Tregs) and activated CD4^+^ T cells, alongside depletion of mast cells and eosinophils (Figures 5H, 6H). This specific immune contexture may explain the observed correlation with poor immunotherapy response, as Tregs are known to suppress anti-tumor immunity while mast cells/eosinophils often associate with favorable outcomes. Interestingly, Our single-cell RNA-seq analysis of clinical samples (GSE241934) revealed an intriguing paradox: these genes were paradoxically downregulated in EGFR-mutant tumors compared to the upregulation observed in resistant cell lines (Figures 7A–F). This divergence likely reflects fundamental biological differences between *in vitro* models and clinical samples. *In vitro* models capture acquired resistance through direct drug selection pressure, where these genes may confer survival advantages. However, clinical samples represent a more complex ecosystem, as the patients in the GSE241934 dataset underwent neoadjuvant immunochemotherapy, introducing additional variables that differentiate them from *in vitro* models. A key finding from our single-cell validation was that the prognostic power of our signature persisted in a cohort uniformly treated with neoadjuvant immunochemotherapy. Crucially, a sensitivity analysis revealed that the signature score was not associated with MPR status. This dissociation is highly informative: it suggests that the aggressive biology captured by our drug resistance-derived signature operates through mechanisms distinct from those determining immediate response to immunochemotherapy. It may instead reflect a tumor’s inherent capacity for long-term progression, immune evasion, or relapse, explaining its stable prognostic value across different clinical contexts. A limitation of our study is the use of a single-cell cohort where all patients received neoadjuvant therapy. However, our analysis showing no link between the signature and MPR status strengthens our confidence that the prognostic signal is genuine and not merely a reflection of treatment response. Our study clarifies the clinical applicability of the 3-gene signature. While the genes were identified in a model of drug resistance, the integrated RiskScore functions primarily as a prognostic, not a predictive, biomarker. This is evidenced by our finding that the score did not predict pathological response to neoadjuvant immunochemotherapy in a clinical cohort. This distinction is critical: our signature identifies a patient subgroup with aggressive tumor biology and poor survival outcomes, but it does not appear to determine initial response to this specific therapy. Consequently, this signature could be used to stratify high-risk patients who may require more intensive monitoring or alternative therapeutic strategies, rather than to guide the selection of initial immunochemotherapy.

## 5 Conclusion

This research bridges the gap between *in vitro* EGFR-TKI resistance and clinical outcomes in LUAD. We established a RiskScore model using *PPP1R3G*, *CREG2*, and *LYPD3*, validated its predictive accuracy for survival across multiple EGFR-TKIs, and linked it to specific immune microenvironmental characteristics. The identification of distinct immune profiles and the development of a combined nomogram underscore the model’s potential to guide personalized therapy selection, including combination strategies. Despite the need for further experimental confirmation, this work offers a promising tool for improving risk stratification for LUAD patients facing the challenge of EGFR-TKI resistance.

## Data Availability

The data presented in the study are deposited in the Figshare repository, accession link: https://doi.org/10.6084/m9.figshare.28579784.v1
