# Supplementary figures and images for: Prognostic model for lung adenocarcinoma based on experimental drug-resistant cell lines and clinical patients
Source: Front Mol Biosci. 2025 Nov 21;12:1654426. doi: 10.3389/fmolb.2025.1654426 (PMC12678131; doi:10.3389/fmolb.2025.1654426)

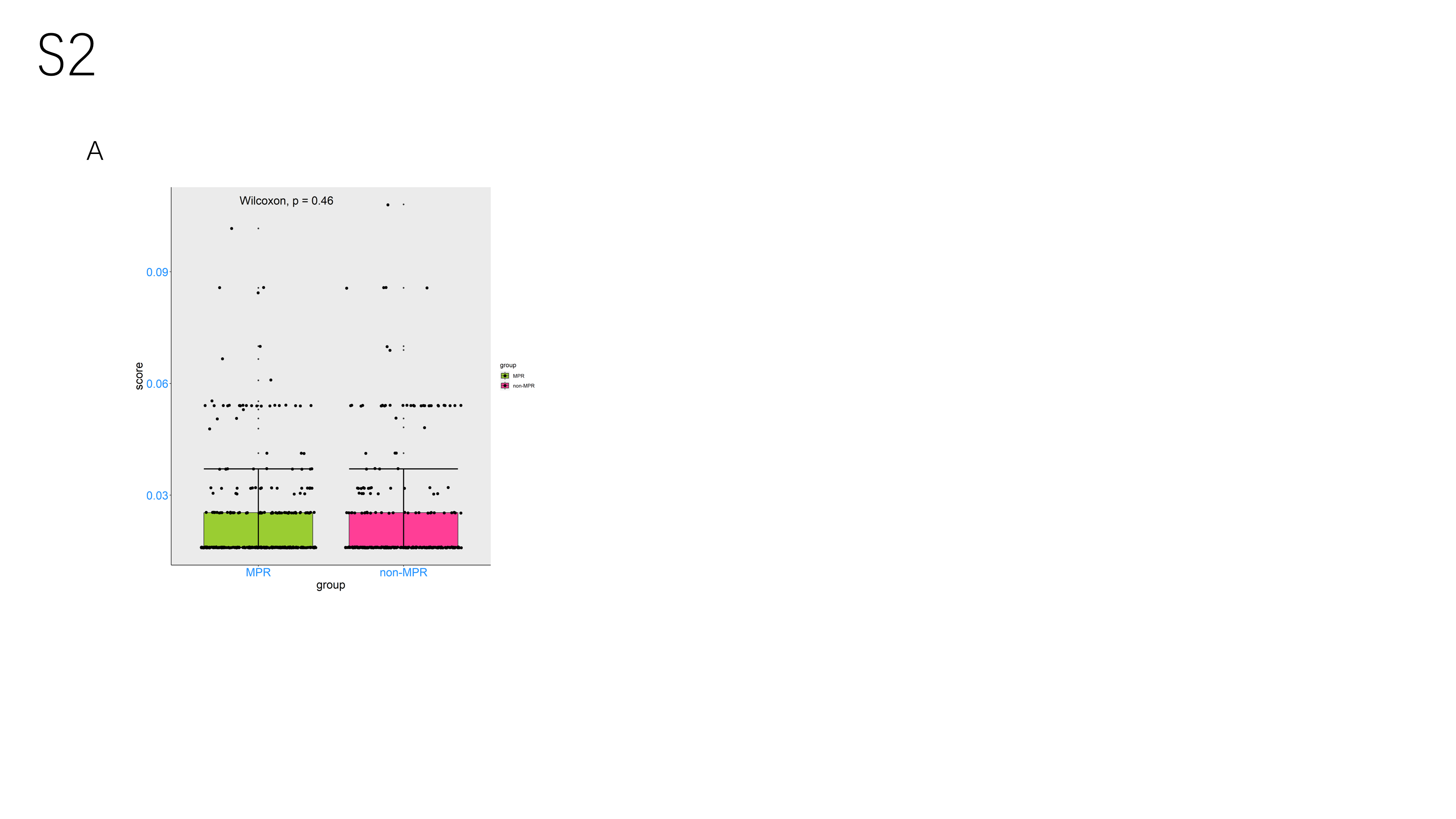

Supplement: Supplementary file 1 [file Image2.tif]

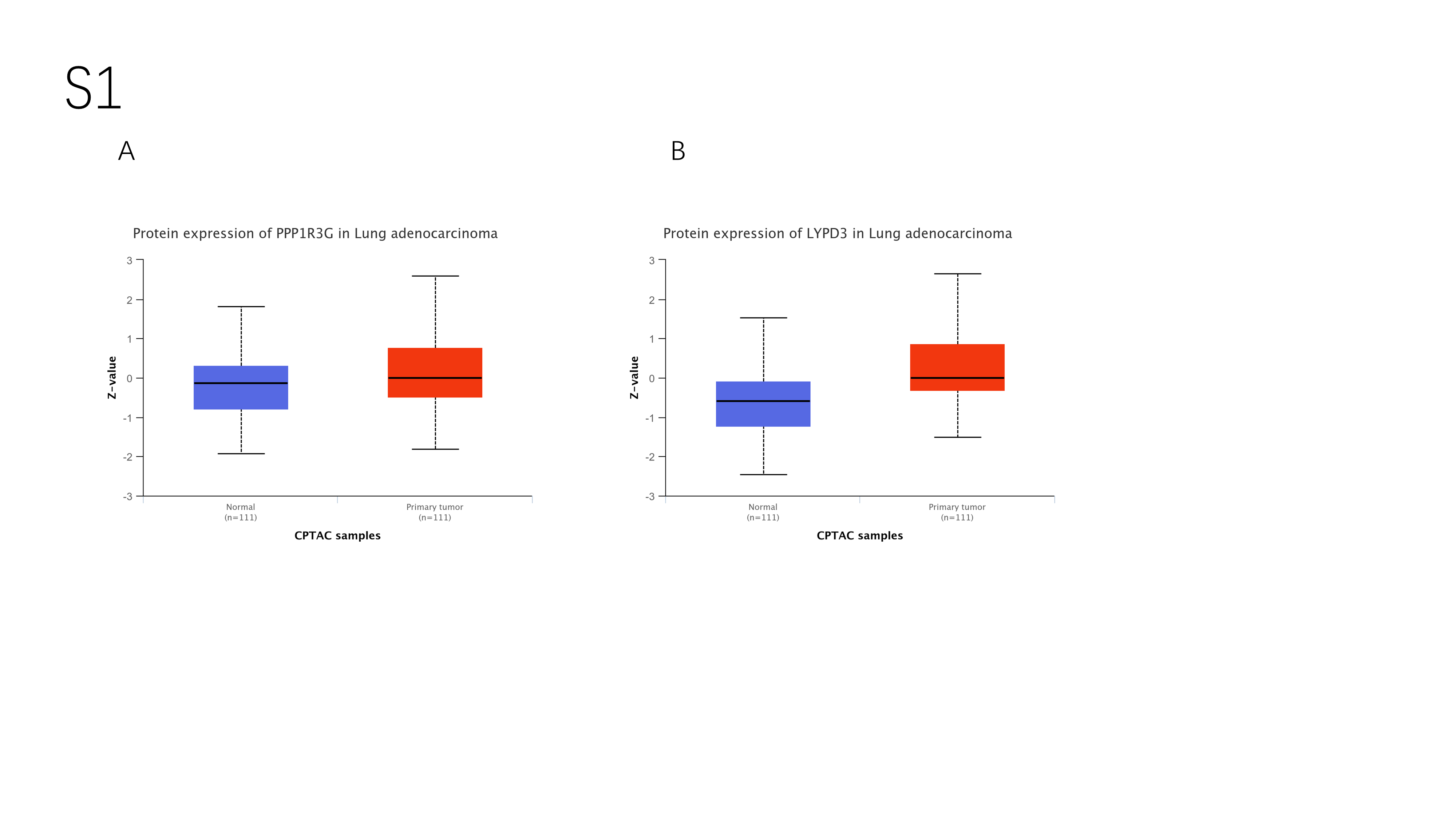

Supplement: Supplementary file 2 [file Image1.tif]
